# Individualised Music Playlist Based on the Iso‐Principle for De‐Escalating Agitation in Older People With Dementia: Protocol for a RCT

**DOI:** 10.1002/nop2.70663

**Published:** 2026-07-03

**Authors:** Daphne Sze Ki Cheung, Jodie Hau Yi Tse, Ken Hok Man Ho, Jennifer Macritchie, Claudia Kam Yuk Lai, Kathleen Buckwalter, Shanshan Wang, Paul Hong Lee, Daniel Bressington, Angel Hiu Tung Chan, Grace Wing Ka Ho

**Affiliations:** ^1^ School of Nursing and Midwifery, Faculty of Health Deakin University Burwood Victoria Australia; ^2^ Deakin Centre for Quality and Patient Safety Research—Bayside Health Partnership Institute for Health Transformation, Deakin University Melbourne Australia; ^3^ School of Nursing The Hong Kong Polytechnic University Hong Kong China; ^4^ School of Nursing and Midwifery La Trobe University Melbourne Australia; ^5^ Department of Music University of Sheffield Sheffield UK; ^6^ The MARCS Institute for Brain, Behaviour and Development, Western Sydney University Sydney Australia; ^7^ College of Nursing University of Iowa Iowa City Iowa USA; ^8^ Southampton Clinical Trials Unit University of Southampton Southampton England UK; ^9^ School of Pharmacy The Chinese University of Hong Kong Hong Kong China; ^10^ School of Nursing Chiang Mai University Chiang Mai Thailand

**Keywords:** aggression, agitation, carer, de‐escalation, dementia, distress, music

## Abstract

**Aim:**

To evaluate the efficacy of an individualised music playlist composed of preferred music genres sequenced using the Iso‐Principle (InMP) for immediate de‐escalation of agitation in dementia, compared with preferred music (PM) and treatment as usual (TAU).

**Design:**

A multi‐centre, three‐arm randomised controlled trial.

**Methods:**

Residential home residents with dementia and significant agitation would be randomly allocated to InMP, PM, or TAU groups. All participants would receive usual de‐escalation techniques when agitated. The InMP group would additionally listen to music sequenced by the Iso‐Principle for 30 min when agitation begins, while the PM group would listen to randomly sequenced preferred music. The primary outcome is agitation level, assessed every 5 min for an hour after each agitation onset. Scores from each 5‐min interval would be calculated to give an area‐under‐curve score to summarise the agitation level of that episode and compared across groups. Secondary outcomes include agitation frequency, behavioural and psychological symptoms severity and carer distress, measured at baseline and Week 6 using CMAI and NPI‐Q and analysed using GEE. Conventional content analysis of semi‐structured interviews with care staff would be used for implementation factors.

**Implications for the Profession and/or Patient Care:**

This study would employ a rigorous approach to evaluate the effects of the intervention. The findings would inform evidence‐based strategies for nursing practice to promptly de‐escalate agitation in dementia, potentially reducing reliance on medications and physical restraints, improving care quality, and alleviating carer burden.

**Impact:**

Agitation negatively impacts both residents and carers. While music interventions can reduce agitation frequency, their effectiveness for immediate de‐escalation and impact on carer distress remain unknown. This study addresses the knowledge gap and aims to provide practical solutions for a prevalent clinical problem.

**Patient or Public Contribution:**

Service providers commented on intervention design and outcome measurement approaches, ensuring the research addresses the needs of individuals with dementia and the protocol is practical for implementation.

**Trial Registration:**

ClinicalTrials.gov: NCT06104436

## 1 Introduction

Dementia is a neurocognitive disorder that affected more than 57 million individuals worldwide in 2021 (World Health Organization 2025), 56.4%–74.6% of whom living in residential facilities exhibited agitation (Halpern et al. 2019; Palm et al. 2018). Agitation in individuals with dementia are characterised by a range of behaviours, such as restlessness and verbal or physical aggression, with emotional distress and excessive dependence in the activities of daily living (Cummings et al. 2015). Agitation is associated with adverse outcomes for people with dementia and their carers. Agitation increases the odds of psychotropic medication prescription (Brimelow et al. 2019) and physical restraints (Andres et al. 2024). More frequent agitation is correlated with lower quality of life among nursing home residents with dementia (Schmüdderich et al. 2021) and is associated with staff burnout (Yan et al. 2025). Furthermore, the healthcare costs in the agitation cohort were found 57% higher than no agitation counterpart (Grossberg et al. 2024). It is essential to identify effective and acceptable approaches to alleviate this problem.

## 2 Background

To calm people with dementia who are agitated, pharmacological and non‐pharmacological approaches are available. Pharmacological approaches are not recommended as first‐line approaches due to their uncertain efficacy and safety (Kongpakwattana et al. 2018). International clinical practice guidelines recommend non‐pharmacological approaches as first‐line treatments (National Health Service 2022; The Royal Australian College of General Practitioners 2020). Calming an agitated person with dementia should begin with ensuring safety and identifying the triggers; however, communication challenges with these individuals often make it difficult to determine the underlying causes of their agitation (Mulkey and Munro 2021). A systematic review has found that certain sensory‐based interventions, particularly music‐related ones, appeared to be effective in de‐escalating agitation within 15 min (Cheung, Wang, et al. 2023b). These findings suggest that music‐related interventions can be helpful adjuncts in calming people with dementia who are experiencing agitation.

According to the Progressively Lowered Stress Threshold Model, people with dementia have a lowered stress threshold, which makes them generally more vulnerable to stressors (Hall and Buckwalter 1987). This vulnerability manifests through their diminished ability to receive, process and respond to environmental stimuli that might not affect those without cognitive impairment (Cheung et al. 2011; Hall and Buckwalter 1987). Therefore, reducing their perceived stress would result in the de‐escalation of agitation as a manifestation of stress. Music might distract an individual by muffling noise in the environment and divert feelings of anxiety to a more positive, stress‐free experience (Cooke et al. 2005). While music's calming effect is recognised, important questions remain about which specific types of music are most effective for de‐escalating agitation in persons with dementia.

There are two main types of music being used for managing agitation in dementia: one relies on calming music, and the other involves playing preferred music (Cheung, Wang, et al. 2023b). Calming, or soothing music, is typically characterised by a slow tempo and specific qualities such as gentle melodies and minimal rhythmic complexity (e.g., soft classical music like *Canon in D*). However, studies have shown mixed results with this approach; for instance, Gerdner (2000) found that relaxing classical music did not effectively reduce agitation. On the other hand, preferred music, which is tied to an individual's positive memories and emotions, is considered more appropriate in alleviating agitation. This is because it can evoke positive emotional responses and foster a sense of familiarity and comfort (Kelly et al. 2023).

In addition to using pre‐recorded music, a promising approach commonly employed in music therapy is the Iso‐principle. This principle requires the therapist to manipulate the mood of clients systematically by gradually shifting the musical elements through playing live music, such as rhythm, tempo, melody, harmony, and mood of the music (Starcke et al. 2021). The sequence in the use of music follows three steps: (a) First, play a piece of music that captures the attention of the client and fosters listening to therapeutic music. (b) Next, play music with a rhythm that matches the client's internal state—slower rhythms for depressed clients and faster rhythms for people experiencing manic states. (c) Finally, gradually shift the client's internal state in a more positive direction by manipulating the musical elements (Starcke et al. 2021). The Iso‐Principle aligns with the Entrainment Theory, a process to synchronise internal body rhythm, such as respiration, heart rate and blood pressure, with external musical rhythm (Kim et al. 2018). These biological oscillators might reflect the stress of an individual and be correlated with agitation in dementia (Ellis et al. 2012; Spasojevic et al. 2021).

There is empirical evidence supporting the entrainment effects of music on physiological stress parameters. For example, by adjusting the musical parameters such as tempo, the heart rate and respiration of listeners can be entrained and altered accordingly, leading to relaxation and relief from depressive symptoms (Bretherton et al. 2019; Ellis et al. 2012; Heiderscheit and Madson 2015). Music listening interventions based on Iso‐Principle have high potential to de‐escalate agitation in people with dementia, supported by the Progressive Lowered Stress Threshold Model and the Entrainment Theory. Our team has conducted a feasibility study on using an individualised music playlist based on Iso‐Principle for de‐escalating agitation of people with dementia (Cheung et al. 2025). It demonstrated that the intervention is welcomed by staff and older adults with dementia.

## 3 The Study

This study builds upon our feasibility study and aims to investigate the effects of an individualised music playlist composed of preferred music sequenced according to the Iso‐Principle on agitation in people with dementia and its impact on their formal carers in residential care homes. The research questions are as below.

### 3.1 Primary Research Question (RQ1)

What are the effects of an individualised music playlist composed of preferred music, sequenced according to the Iso‐Principle (hereafter, referred to as InMP), on the immediate de‐escalation of agitation in nursing home residents with dementia, as compared to preferred music (PM) and treatment as usual (TAU)?

### 3.2 Secondary Research Questions

What are the effects of the InMP on the agitation occurrence frequency and behavioural and psychological symptoms of dementia (BPSD) severity after 6 weeks, as compared to PM and TAU?

What are the effects of the InMP on formal carer distress related to agitation after 6 weeks, as compared to PM and TAU?

What are the challenges and enablers when implementing the InMP from the perspective of formal carers?

## 4 Methods

### 4.1 Design and Participant Timeline

**FIGURE 1:**
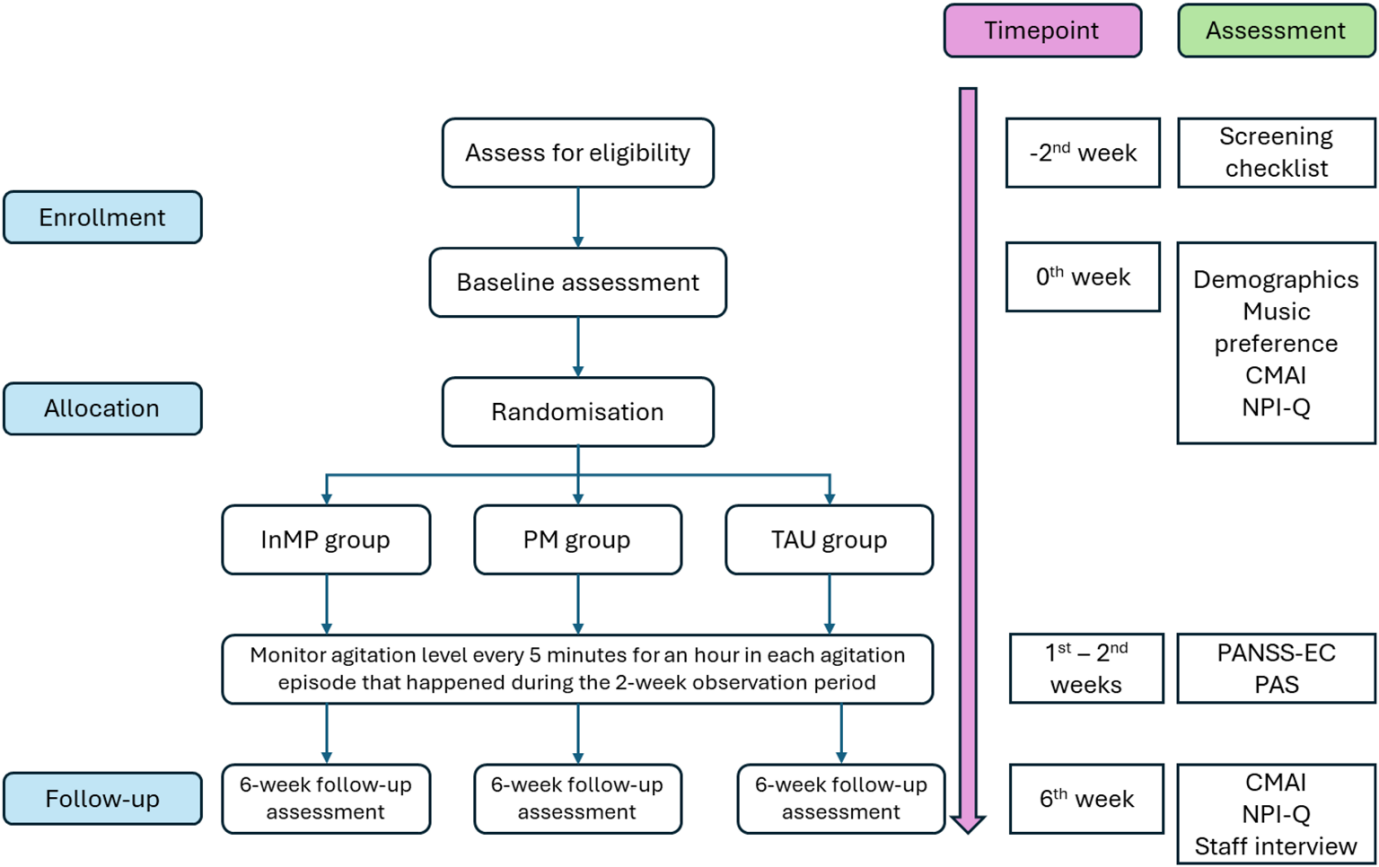
Study flow chart. CMAI, Cohen–Mansfield Agitation Inventory for agitation occurrence frequency; NPI‐Q, Neuropsychiatric Inventory Questionnaire for carer's distress related to behavioural and psychological symptoms; PANSS‐EC, the Excited Component of the Positive and Negative Syndrome Scale for the level of agitation; PAS, Pittsburgh Agitation Scale for the level of agitation.

This is a multi‐centre, three‐parallel arms randomised controlled trial. The trial protocol was prospectively registered on ClinicalTrials.gov (NCT06104436) on 26 Oct 2023. Eligible participants who agree to participate and with the consent form signed by proxy would be randomly allocated to InMP (experimental group), PM (comparison group), or TAU (control group) at 1:1:1 ratio. The study would be conducted over 6 weeks, and the intervention or control conditions would be given whenever there is an agitation episode (see Figure 1). A 6‐week study period is set because in a previous randomised controlled trial, the frequency of agitation occurrence in the preferred music listening group was significantly reduced after 6 weeks (Cheung et al. 2020). It is assumed that a significant reduction in agitation occurrence frequency (i.e., secondary outcome) would be at least achieved. This protocol is reported according to the Standard Protocol Items: Recommendations for International Trials (SPIRIT) checklist (Chan et al. 2013) (see Data S1).

### 4.2 Participants, Interventions, and Outcomes

#### 4.2.1 Setting and Participants

Participants would be recruited from residential care homes for the older adults in Hong Kong. Inclusion criteria are those:

- with a medical diagnosis of any type of dementia;
- presented with significant agitation in the previous 2 weeks before recruitment. It means a Cohen–Mansfield Agitation Inventory (CMAI) total score > 39 at baseline, a common cut‐off score in the other relevant local study (Yuen and Kwok 2019); and
- expected to reside in the facility during the study period, as otherwise observation of outcomes cannot be carried out.

Exclusion criteria are residents who:

- have been admitted to the facility for less than 3 months because they would still be adapting to their new environment;
- are participating in other studies or experimental therapies that may confound the results;
- have an unstable comorbid psychiatric illness or condition such as depression, schizophrenia, or delirium, who may need specific attention in the music prescription;
- have acute physical illnesses or symptoms that may induce agitation, such as unmanaged pain or urinary tract infection; or
- have an uncorrectable hearing impairment; or
- have a known history of agitation triggered by wearing headphones, neckband, or participating in any music intervention.

Residents who are receiving anti‐psychotropic medications would be allowed to participate, but any change in the prescriptions or new prescriptions would be monitored. Sensitivity analyses would be conducted to examine the effects among those who have or have not been prescribed antipsychotics for managing agitation.

#### 4.2.2 Intervention and Control Conditions

Participants would be randomised into one of the three groups: InMP, PM and TAU (see Table 1). Interventions would be delivered individually according to the group assignment over 6 weeks, when the participant exhibits clinically moderate to severe agitation (defined as a total score of the Excited Component of the Positive and Negative Syndrome Scale (PANSS‐EC) of ≥ 17). Although a score of 14 is generally regarded as the minimum threshold for clinically significant agitation and a score of 20 or higher indicates severe agitation (Pompili et al. 2021), we selected 17 as the cut‐off for intervention. This decision was informed by our experience in the feasibility trial, ensuring that symptoms are sufficiently pronounced for frontline care staff (hereafter, named as formal carer) to identify in a busy healthcare setting, while still manageable without the need for urgent or invasive measures. Each participant would receive their usual care during the study period, including assistance with the activities of daily living.

**TABLE 1:** The active components of the intervention and control conditions.

	InMP	PM	TAU
Treatment as usual	✓	✓	✓
Preferred music listening (during the episodes of agitation and in the morning twice weekly for 6 weeks)	✓	✓	
Music sequenced according to Iso‐Principle	✓		

Formal carers, including nurses, personal care workers and other allied health professionals, are deemed most suitable to provide the intervention or control conditions as they work closely with residents and can best identify early signs of agitation. A 1‐hour training session would be given to them for administering the interventions. The training would cover the major topics, such as accurately identifying overt activity that reflects the beginning of an episode of agitation; how to operate the music playing devices; and the intervention regime. Competency would be assessed through direct observation using a standardised checklist before staff are requested to deliver the intervention or control condition independently. An Operation Manual would be provided to them, and they would be asked to record the date and time of agitation throughout the 6‐week study period. Additional instructions would be provided to those showing difficulties. Telephone follow‐ups or site visits to the residential care homes would be arranged to evaluate formal carers' adherence to the protocol, explore their implementation barriers and provide additional instructions.

##### 4.2.2.1 Individualised Music Playlist (InMP) Group

Participants in the InMP group would be prescribed a 30‐mins individualised playlist with preferred music genres sequenced by a registered music therapist according to the Iso‐Principle. The music therapist selected 50 music pieces favoured by most older adults in Hong Kong, primarily songs from 1940 to 1980, along with some instrumental and religious music. Participants, family members, and formal carers would be invited to identify 10–12 pieces of music selected from the list or to suggest additional preferred tracks, during baseline data collection. The therapist would then organise these selections into a 30‐min playlist based on arousal properties (e.g., from stimulating to relaxing), emotional quality (e.g., uplifting to peaceful), and structural features (e.g., from fast tempo to slow tempo) (Cheung et al. 2025). The playlists would be modified upon the participant's request or if they are observed by the trial coordinator as not suitable after consulting the music therapist (e.g., the agitation is worsened, or the emotion of the participant is turning sad). The adjustment would usually take place in the first week. The sequence of, and the selected music genres being played would be constant throughout the 6 weeks of intervention.

The music playlist would be 30 min in duration because previous studies have found that the duration is acceptable and optimal for the participants in terms of immediately de‐escalating during the episode of agitation back to a desirable level and reducing their agitation occurrence frequency after 6 weeks (Cheung et al. 2020, 2025).

The prescribed music would be delivered with a wireless neckband speaker connected to an iPod Touch, subscribed to YouTube Premium for ad‐free and offline music. The neckband speaker (Sony Wireless Neckband Speaker SRS‐NS7) weighs approximately 318 g, which allows the participant to be immersed in the music environment without wearing a headphone, which is well accepted by participants during agitation in our feasibility study (Cheung et al. 2025). To increase the familiarity of the music playlist and comfort with listening to music with the neckband speaker, they would listen to the prescribed playlists in the morning after breakfast, sitting in a quiet corner of the residential care home, twice weekly for 6 weeks.

##### 4.2.2.2 Preferred Music (PM) Group

To help determine if any agitation de‐escalation effect comes from the sequence based on Iso‐Principle or just from listening to preferred music in any order, a PM group is designed. Participants in the PM group would instead listen to their preferred music genres played in a random sequence while the duration of the playlists, mode of delivery of the intervention and regular music listening sessions remain the same as those for the InMP group. The preferred music genres are identified using the same approach as is used in the InMP group.

##### 4.2.2.3 Treatment as Usual (TAU) Group

Designing a TAU group serves two purposes: maintaining ethical standards by ensuring the control group participants receive established care; and allowing measurement of whether music listening provides benefits beyond existing practices. The participants in the TAU group would wear a silent neckband speaker for 30 min. The usual treatments for de‐escalation include addressing potential known physical and environmental triggers of agitation, such as pain or hunger, or high room temperatures. This includes providing prescribed pain relief, offering food or drinks, or removing extra layers of clothing. The comparison among three groups is shown in Table 1.

#### 4.2.3 Outcomes

The outcome assessment plan is illustrated in Figure 1.

To address RQ1 about the effects of InMP for immediately de‐escalating agitation as compared with PM and TAU, the primary outcome agitation severity would be measured with the Pittsburgh Agitation Scale (PAS). While the PANSS‐EC is commonly being used in antipsychotic drug trials for assessing agitated patients, it would also be used in this study to supplement the evaluation.

The PAS consists of four items—aberrant vocalisation, motor agitation, aggressiveness, and resistiveness to care, with the item‐rating ranging from 0 (not present) to 4 (extreme). It has been validated among people with dementia with good inter‐rater reliability (ICC = 0.82) (Rosen et al. 1994). The PANSS‐EC consists of five items—excitement, hostility, tension, uncooperativeness, and poor impulse control, rating from 1 (absent) to 7 (extreme). It has good internal consistency (*α* = 0.86) (Montoya et al. 2011). The observations would be conducted and rated by research assistants (RA) who are trained to be consistent with the first author, giving the ICC of 0.9 or above.

To address RQ 2 and RQ3 about the effects of InMP for reducing the agitation occurrence frequency, BPSD severity, and related carer's distress upon the completion of the 6‐week intervention, the Cohen–Mansfield Agitation Inventory (CMAI) and Neuropsychiatry Inventory—Questionnaire (NPI‐Q) would be used rated by a formal carer.

The CMAI is a 29‐item scale with a 7‐point rating of agitation frequency ranging from 1 (never) to 7 (several times an hour) over the past 2 weeks. It is commonly used in research about dementia agitation, and it demonstrates acceptable internal consistency (*α* = 0.76) and inter‐rater reliability (*r* = 0.88–0.92)(Cohen‐Mansfield 1986). The NPI‐Q evaluates the presence, severity and associated carer distress across 12 BPSD, such as delusions, depression, and appetite. For each symptom, the formal carer would indicate its presence (1 for yes/0 for no), would rate its severity on a 3‐point scale (1 = mild to 3 = severe), and the associated carer distress on a 5‐point scale (0 = no distress to 5 = extreme distress). It has demonstrated acceptable internal consistency (*α* = 0.756) and test–retest reliability (ICC = 0.990) (Wong et al. 2014).

To address RQ4 about the challenges and enablers in the implementation of the InMP from the perspective of formal carers, semi‐structured individual interviews or focus groups would be used. Interviews would be audio/video recorded and transcribed verbatim for analysis.

#### 4.2.4 Sample Size

To address the primary RQ, the total sample size required for this study is estimated to be 159 episodes of agitation. By assuming that each participant with a CMAI total score > 39 would have at least two episodes over the 2‐weeks observation period, 81 participants would be required. The required number of participants was calculated with G*Power 3.1, based on the following assumption: *α* = 0.05, power = 0.8, at Cohen's *f* = 0.25 (medium effect size informed by the feasibility study; Cheung et al. 2025).

#### 4.2.5 Recruitment and Retention

An invitation letter would be sent to all residential care homes for the older adults in Hong Kong with a capacity of 100 places or above. These homes are named as Care and Attention Home in Hong Kong, which provide residential care, meals, personal care and nursing care for older adults who are in poor health with a deficiency in activities of daily living. During the recruitment process, potential participants would be referred to the research team by the responsible staff of the participating homes after obtaining the initial consent from the proxy. The research team members would review their medical records and their formal carers' or proxies' reports to determine their eligibility for participation in this study.

Several strategies would be employed to maximise participant retention. The study design minimises burden on participants by using observational measures rather than requiring direct interviews (Cheung et al. 2021). Regular communication with the proxies (usually a family member), who provide consent, would be maintained throughout the study period to address any concerns promptly. Engagement with formal carers and administrators would be fostered through regular meetings and ongoing implementation support.

### 4.3 Assignment of Intervention or Control Conditions

#### 4.3.1 Allocation

Randomisation would be conducted using the online randomisation service Sealed Envelope. After screening and collecting baseline data, eligible participants would be allocated by Sealed Envelope to one of the three arms (InMP, PM, or TAU group) at a 1:1:1 ratio using permuted block randomisation with a block size of 3. This approach enhances allocation concealment while maximising the possibility of achieving a balanced group size. To minimise the potential of selection bias, the randomisation sequence would be generated by an independent RA not involved in participant recruitment or assessment and concealed from all staff involved in participant recruitment and assessment. After confirming eligibility, only the trial coordinator would access the secure online system to obtain the allocation, and create the playlist (InMP, PM or silent) accordingly for later implementation by a frontline care staff.

#### 4.3.2 Blinding

Due to the nature of the intervention, complete participant blinding is not feasible, as participants are aware of the sound delivered through the neckband speaker. To address the potential investigator bias, we would implement comprehensive blinding strategies. Formal carers delivering the intervention and rating the agitation frequency and other behavioural and psychological symptoms at baseline and Week 6, would be purposively trained but remain unaware of group assignments. Each playlist (either InMP, PM, or silent for TAU group) would be prepared by a trial coordinator not involved in outcome assessment and labelled only with the participant's name. The first 30 s of the audio script would inform participants that the audio would last approximately 30 min and are designed to minimise the likelihood of formal carers identifying the group allocation while adjusting the volume. Trained RA who would observe the agitation intensity, would be blinded to group allocation, and they would only observe from a distance where they cannot hear the neckband speaker output.

### 4.4 Data Collection, Management, and Analysis

#### 4.4.1 Data Collection

The data collected at each time point are summarised in Table 2. During the first 2 weeks of the study, agitation episodes would be intensively monitored by the RA. Based on our feasibility study, participants typically show a noticeable reduction in agitation frequency after engaging with the intervention. With a CMAI total score > 39, we anticipate each participant would experience at least two agitation episodes during the 2‐week observation period, providing sufficient data for analysis while avoiding unnecessary extension of the timeframe.

**TABLE 2:** Data collection timepoints.

Target group	Outcomes	Approach	Baseline	Weeks 1–2	Week 6
Participant	Demographics	Questionnaire	X		
	Music preference		X		
	PAS	Rated by RA for each observed agitation episode		X	
	PANSS‐EC			X	
	CMAI	Rated by a formal carer	X		X
Participant and frontline staff	NPI‐Q		X		X
Frontline staff	Implementation	Semi‐structured interview			X

Abbreviations: CMAI, Cohen–Mansfield Agitation Scale; NPI‐Q, Neuropsychiatric Inventory—Questionnaire; PANSS‐EC, Positive and Negative Syndrome Scale—Excited Component; PAS, Pittsburgh Agitation Scale.

All numerical data would be collected electronically using Qualtrics. Trained RA would intensively observe and rate the agitation intensity via PAS and PANSS‐EC every 5 min for 1 h following the onset of each agitation episode requiring intervention during the 8 a.m. to 6 p.m. Monday—Sunday observation window. This timeframe is established in consultation with residential care facility administrators to ensure feasibility within their operational constraints. One trained RA would be responsible for observing at most six participants in each residential care homes. In facilities with more than six participants or higher frequency of agitation episodes, additional RA would be assigned to ensure comprehensive coverage and timely assessment. We would also invite the formal carers to report the frequency of agitation and other BPSD at baseline and at the 6th week.

Semi‐structured interviews with formal carers would be conducted to explore implementation factors, challenges and facilitators following the final data collection and the disclosure of group allocation. The respondents would have been involved in intervention delivery. The interviews would take place via videoconferencing, a method preferred by busy formal carers for its convenience. The prompting questions are: (1) What were the behaviours of older adults in the InMP group during the intervention? (2) What are the changes during and after listening? (3) What do you think about the intervention being integrated into routine practice? (4) How to improve the effects? (5) Any comments on the intervention implementation? All interviews would be video‐/audio‐recorded and transcribed for analysis.

#### 4.4.2 Data Management

To preserve confidentiality, each participant would be assigned a unique identifier, which would be used on all data collection forms. A secure master identification log linking participant identities to study IDs would be accessible only to the trial coordinator and principal investigator. All analyses and reporting would use anonymised datasets to protect participant confidentiality. Data would be entered directly by the assessor using Qualtrics with programmed validation rules to flag missing values, out‐of‐range entries, and logical inconsistencies in real‐time. There is no plan for data sharing.

#### 4.4.3 Statistical Methods

Statistical analyses would be performed using IBM SPSS Statistics 30 (IBM Corp 2024) or the most updated version that the investigators have access to at the time of data analysis. Demographic and clinical characteristics would be summarised using means and standard deviations for continuous variables, or otherwise as frequencies and percentages for categorical variables. Data normality would be assessed using the Shapiro–Wilk test while Levene's test would evaluate homogeneity of variance. Baseline comparisons across the three groups would employ one‐way ANOVA for normally distributed continuous variables (or Kruskal–Wallis tests if parametric assumptions are violated) and a Chi‐squared test for categorical variables.

Intention‐to‐treat approach would be employed in all statistical analysis with all participants after randomisation would be analysed. The primary outcome (i.e., level of agitation every 5 min after implementing the interventions once the agitation episode begins) would be analysed using an analysis of serial measurements utilising the area under the curve (AUC), capturing the integrated response over time rather than isolated timepoints. Between group differences in AUC would be assessed using ANCOVA, adjusting for baseline agitation scores. Secondary outcomes (i.e., agitation occurrence frequency, BPSD severity, and carer's distress related to agitation), would be analysed using a generalised estimating equation (GEE). Missing data would not be imputed as GEE allows missing data. Statistical significance would be based on *p* < 0.05 for all statistical analyses.

#### 4.4.4 Qualitative Analysis

The interview transcripts would be analysed with conventional content analysis (Hsieh and Shannon 2005) to explore the implementation challenges and facilitators within the long‐term care setting. The analytical process would begin by reading the data repeatedly to achieve immersion and a sense of whole the implementation of the intervention by two researchers. Then codes would be developed and clustered to form category of facilitators and challenges of the implementation by the two authors and be discussed and finalised with other team members. Verbatim transcription and content analysis would be managed by NVivo version 15 (Lumivero 2024).

### 4.5 Monitoring

The daily operation of the trial would be overseen by the principal investigator and the trial coordinator. The team would have responsibility for ensuring the compliance and progress of the study in relation to regulatory, administrative, industry and safety issues. Regular meetings would take place during the study. All adverse events would be recorded and reported to the IRB, study sites, and participants' families.

### 4.6 Ethical Considerations

Ethical approval has been obtained from the Institutional Review Board at the Hong Kong Polytechnic University (HSEARS20220107003). The research team will adhere to the Declaration of Helsinki, the Good Clinical Practice guidelines and their subsequent updates for research involving human participants. Access approval would also be obtained from the participating study sites before the participants are recruited. Proxies and participants would be informed about the possible risks and benefits of participation. Participation would be voluntary, and the participants would be free to withdraw at any time. A written information sheet containing the above information would be given to all participants and their proxies. As participants with dementia may lack the capacity to consent, assent would be sought, and an appropriate consultee would be asked for an opinion on the individual's involvement if the proxy is absent. Assent would be periodically reassessed. Procedural consent would also be sought. According to the literature and our past experience, music interventions are safe, and no adverse reactions have been reported. All data would be securely stored and would be destroyed 7 years after the publication of the trial results to protect the confidentiality of participants.

## 5 Discussion

Given the rising prevalence of dementia and the high incidence of agitation in people with dementia, it is imperative to provide an effective non‐pharmacological intervention to promptly de‐escalate agitated behaviours. To our best knowledge, this is the first full‐scale RCT evaluating the effects of a music playlist designed based on the Iso‐Principle in de‐escalating agitation among older adults with dementia. Most studies to date focus on the effects of preferred music on agitation frequency in older adults with dementia. While anecdotal reports suggest that favourite music may have a calming effect, rigorous evaluation is lacking, and the appropriateness of music for agitation episodes remains unclear. This study would offer valuable insights into the immediate de‐escalation effects of listening to preferred music sequenced according to the Iso‐Principle and enhance our understanding of the therapeutic value of music through rigorous research. The findings would have significant implications for nursing practice in dementia care, particularly for agitation management.

Currently, nursing interventions for agitation often rely on verbally comforting an agitated older adult with dementia, identifying triggers, or employing distraction techniques (James et al. 2023), which may not always be effective and can be inefficient because of the diminished verbal communication ability. A recent study also highlighted the eagerness of practitioners for getting specific about how non‐pharmacological intervention can be implemented (Gray et al. 2022). This study aims to equip nurses and other carers with evidence‐based, practical skills to de‐escalate agitation more effectively, potentially reducing the use of pharmacological interventions and physical restraints, improving care quality, and alleviating carer distress.

Moreover, our multi‐method approach, collecting both quantitative and qualitative data, would provide a comprehensive understanding of the intervention's effects, implementation challenges, and facilitating factors. This holistic perspective is essential for translating findings into clinical nursing practice and would inform future implementation studies (Cheung, Ho, et al. 2023a). In addition, this study would not only evaluate the effects of the intervention on participants with dementia but also examine its impact on the distress experienced by formal carers. A review of reviews highlighted that current evidence largely overlooks the effects of non‐pharmacological interventions on direct care workers (Karlsen et al. 2023). Our study would address this critical knowledge gap.

This intervention offers significant nursing implications in residential care settings. If proven effective, this approach could be incorporated into nursing care plans as a first‐line response to early signs of agitation, potentially reducing reliance on PRN medications and promoting person‐centred care. Implementation would require minimal training for nursing staff, focusing on agitation recognition and basic technical skills related to the music playing devices. Additionally, the intervention aligns with nursing's holistic care by addressing emotional and psychological needs rather than merely managing symptoms, thus potentially improving the overall care quality.

### 5.1 Potential Limitations

This proposed study is not without potential limitations. Although we attempt to blind the assessor (RA who would rate the state of agitation, the formal carers who would rate the frequency of agitation and BPSD), as well as the formal carers who would provide the intervention or control conditions about the group assignment, the assignment might be inevitably known because some participants might sing, clap or tap their feet while listening, which was observed in our feasibility study (Cheung et al. 2025). Additionally, the study is conducted in a specific cultural context in Hong Kong, which may limit generalisability to other cultural settings where music preferences and care practices may differ. Future research should consider conducting multi‐cultural trials to address this limitation.

## 6 Conclusion

This randomised controlled trial would provide important evidence on the effectiveness of individualised music playlists based on the Iso‐Principle for de‐escalating agitation in people with dementia. If effective, this intervention offers a practical, non‐pharmacological approach that can be readily implemented by nursing staff in residential care settings. The findings would contribute to evidence‐based nursing practice for managing one of the most challenging aspects of dementia care, potentially improving quality of life for both residents and their carers.

## 7 Relevance to Clinical Practice

Music listening interventions have been demonstrated to be effective in preventing agitation among individuals with dementia and are feasible across diverse care settings. Their potential to calm an agitated individual with dementia is gaining increasing attention within the field. This trial will employ an intensive and innovative approach to compare the effects of different types of music playlists against usual care. Nurses, who are consistently at the forefront of dementia care, play a critical role in managing its associated symptoms. The findings from this trial will contribute to evidence‐based practice and support the implementation of person‐centred care strategies.

## Author Contributions

Conceptualisation: Daphne Sze Ki Cheung, Ken Hok Man Ho, Jennifer Macritchie, Claudia Kam Yuk Lai, Kathleen Buckwalter, Shanshan Wang, and Daniel Bressington. Data curation: Daphne Sze Ki Cheung, Jodie Hau Yi Tse, Ken Hok Man Ho, Paul Hong Lee, and Angel Hiu Tung Chan. Formal analysis: Daphne Sze Ki Cheung, Ken Hok Man Ho, Jodie Hau Yi Tse, and Paul Hong Lee. Funding acquisition: Daphne Sze Ki Cheung, Ken Hok Man Ho, Jennifer Macritchie, Claudia Kam Yuk Lai, Kathleen Buckwalter, Shanshan Wang, Paul Hong Lee, Daniel Bressington, and Grace Wing Ka Ho. Methodology: Daphne Sze Ki Cheung, Jodie Hau Yi Tse, Ken Hok Man Ho, Jennifer Macritchie, Claudia Kam Yuk Lai, Kathleen Buckwalter, Shanshan Wang, Paul Hong Lee, Daniel Bressington, and Grace Wing Ka Ho. Project administration: Daphne Sze Ki Cheung, Jodie Hau Yi Tse, Angel Hiu Tung Chan, and Grace Wing Ka Ho. Supervision: Daphne Sze Ki Cheung and Grace Wing Ka Ho. Writing the original draft: Daphne Sze Ki Cheung, Jodie Hau Yi Tse, and Angel Hiu Tung Chan. Reviewing and editing of the draft: all authors.

## Funding

This work was supported by the General Research Fund, Research Grants Council, University Grants Committee, Hong Kong (15604923).

## Ethics Statement

This project is approved by the Hong Kong Polytechnic University Human Subjects Ethics Review Committee with the reference number HSEARS20220107003.

## Conflicts of Interest

The authors declare no conflicts of interest.

## Supporting information


**Data S1:** nop270663‐sup‐0001‐Supinfo1.doc.

## Data Availability

The data that support the findings of this study are available on request from the corresponding author. The data are not publicly available due to privacy or ethical restrictions.
